# Collaboration between infection control and occupational health in three continents: a success story with international impact

**DOI:** 10.1186/1472-698X-11-S2-S8

**Published:** 2011-11-08

**Authors:** Annalee Yassi, Elizabeth A Bryce, Jaime Breilh, Marie-Claude Lavoie, Lindiwe Ndelu, Karen Lockhart, Jerry Spiegel

**Affiliations:** 1 University of British Columbia, 2206 East Mall, Vancouver, BC, V6T 1Z3, Canada; 2 Vancouver Coastal Health, 855 West 12th Ave., Vancouver, BC V5Z 1M9, Canada; 3 Universidad Andina Simón Bolívar, Sede Ecuador Toledo N22-80 (Plaza Brasilia), 17-12-569, Quito, Ecuador; 4 Pan American Health Organization, 525 23rd Street NW, Washington, DC, 20037, USA; 5 Medical Bureau of Occupational Disease, 144 De Korte Street, Braamfontein, South Africa

## Abstract

Globalization has been accompanied by the rapid spread of infectious diseases, and further strain on working conditions for health workers globally. Post-SARS, Canadian occupational health and infection control researchers got together to study how to better protect health workers, and found that training was indeed perceived as key to a positive safety culture. This led to developing information and communication technology (ICT) tools. The research conducted also showed the need for better workplace inspections, so a workplace audit tool was also developed to supplement worker questionnaires and the ICT. When invited to join Ecuadorean colleagues to promote occupational health and infection control, these tools were collectively adapted and improved, including face-to-face as well as on-line problem-based learning scenarios. The South African government then invited the team to work with local colleagues to improve occupational health and infection control, resulting in an improved web-based health information system to track incidents, exposures, and occupational injury and diseases. As the H1N1 pandemic struck, the online infection control course was adapted and translated into Spanish, as was a novel skill-building learning tool that permits health workers to practice selecting personal protective equipment. This tool was originally developed in collaboration with the countries from the Caribbean region and the Pan American Health Organization (PAHO). Research from these experiences led to strengthened focus on building capacity of health and safety committees, and new modules are thus being created, informed by that work.

The products developed have been widely heralded as innovative and interactive, leading to their inclusion into “toolkits” used internationally. The tools used in Canada were substantially improved from the collaborative adaptation process for South and Central America and South Africa. This international collaboration between occupational health and infection control researchers led to the improvement of the research framework and development of tools, guidelines and information systems. Furthermore, the research and knowledge-transfer experience highlighted the value of partnership amongst Northern and Southern researchers in terms of sharing resources, experiences and knowledge.

## Background

Working conditions for health workers are undergoing rapid change [1]. New methods for diagnosis and treatment of diseases, combined with rapid communication technology, makes the world’s ability to communicate and disseminate new knowledge remarkably effective; the speed with which the SARS outbreak was controlled [2] and pandemic H1N1 information transmitted are clear illustrations [3]. On the other hand, economic globalization is severely straining healthcare resources, preferentially benefiting richer countries [4,5]. The changes in labour flow[5] and trends to deregulation [4,6] also impact the health and well-being of the labour force. International travel, representing 684 million passengers in 2009 [7], adds complexity in preventing and reducing rapid transmission of infectious diseases across borders.

Rapid travel has intensified the global need for consistent application of infection control principles to ensure the safety of patients, hospital visitors and health workers. Healthcare acquired infections (HAIs) are often linked to invasive devices, longer hospital stays and more time spent in intensive care [8]. These infections make up a substantial proportion of the infectious disease burden in high income as well as in low and middle-income countries (LMICs) [9]. The risk of a HAI is 2–20 times higher in LMICs than in high income countries, and this may be an underestimate, due to differences in the intensity of surveillance [9,10]. An estimated 20% of patients could suffer from preventable HAIs [11]. Along with patients, healthcare workers are also at high risk of exposure to biological agents in healthcare settings [12,13]. Almost half the cases of SARS were in health workers [2], 40% of the hepatitis B and C that occurs in health workers is estimated to be due to occupational exposures [11,14,15], and health workers have a high risk of multiple drug-resistant tuberculosis [16].

To protect the health and safety of patients and health workers in all countries, infection control and occupational health professionals must work closely together. Our interdisciplinary international collaboration has contributed to produce practical tools such as guidelines, on-line and face-to-face training products, checklists, research materials, frameworks and a health information system. This innovative participatory paradigm that has been widely embraced by collaborators and front line health workers.

## The research

Post-SARS epidemic, the Canadian-based team (led by co-authors AY, an occupational health researcher, and EAB, an infection control specialist) conducted research to ascertain the determinants of sustainable adherence to appropriate infection control practices, refining a framework on individual, organizational and environmental factors[17]. In a survey of 1,290 workers across sixteen hospitals in British Columbia the team found that health workers who rated the environmental protective measures highly at their institutions were 12 times more likely to report a high level of compliance with appropriate personal protective practices compared to those who did not rate this factor highly at their institutions. Similarly, those who perceived organizational factors in the workplace to be consistent with safe practices were 10 times more likely to report good compliance. Interestingly, though, there was no association with the individual factors previously thought to be pivotal in affecting compliance [17]. Next, a survey of infection control and occupational health resources and a questionnaire completed by healthcare workers were compared with on-site observational audits in facilities in British Columbia and Ontario. Health workers believed that plans were available to protect against future SARS-like events but audits revealed that these did not exist in many facilities. Both occupational health and infection control were under-resourced post-SARS, with occupational health professionals particularly lacking in British Columbia. There was a discrepancy between health workers’ perception of what was available and what is actually accessible in facilities[18], highlighting the need for better communication.

The findings from our initial research in Canada led to our developing an evidence-based workplace assessment tool. Our initial research in Canada also showed that training health workers was significantly associated with health worker perception of a positive safety culture in their healthcare workplace [17,19]. One of the identified constraints was the limited quantity of information that could be presented at group sessions due to the time restrictions. These sessions were insufficient to build knowledge and good practices on the selection and use of personal protective equipment [20]. These research observations were the impetus for our developing online infection control courses that were self-directed, flexible, interactive, and relevant to day-to-day work activities [21,22].

In the region of the Americas, our team collaborated with the Pan American Health Organization (PAHO) on a project related to the prevention of occupational transmission of infectious diseases among health workers. In collaboration with the Ministry of Health from Ecuador, the team members collectively adapted the Canadian workplace assessment tool and questionnaire to assess knowledge, attitudes and practices in three hospitals in Ecuador (two in Quito and one in the Amazon) [23-25]. The workplace assessment tool comprises a list of occupational hazards, including physical, chemical, biological, ergonomic, safety, and psychological hazards. Under each hazard classification, the evaluator completes the workplace assessment form by indicating whether the environment and practices are satisfactory, require correction but are not an immediate hazard, or require immediate correction. Using the results of the questionnaire and needs assessment, local colleagues identified strengths and challenges at each healthcare facility and initiated projects to address the issues unearthed. For example, campaigns were begun to improve hand hygiene and reduce needlestick injuries, as well as implement much-needed renovations in the emergency department of one of the hospitals [25] – all priorities identified by using the tools developed.

Following the success of this initial work in Canada and Ecuador, the government of the Republic of South Africa (through co-author LN) invited our team to lead a healthy hospital initiative in that country. Again, working closely with local colleagues, we revised the assessment tools, then invited 76 participants to a three-day workshop on occupational health and infection control to complete the initial survey. Invited participants included all the 54 representatives elected from the workforce to serve as health and safety representatives as well as the 16 occupational health and infection control staff members from Pelonomi Hospital, the health facility selected for our pilot study[26]. The participants were then divided into ten groups to conduct workplace audits, covering five domains; Physical Environment, Specific Occupational Health Practices and Hazards, Specific Infection Control Practices, Equipment and Procedures, and Ergonomics. Training sessions were also conducted specifically for medical practitioners, a usually hard-to-reach population, as the Canadian-based research also confirmed[17,19].

Having identified the need for better data collection instruments, we developed the Occupational Health and Safety Information System (OHASIS), a web-based health information system, to track incidents, exposures, risk factors, immunizations and occupational injury and diseases. Based on experience in Canada[27], we ensured that this system particularly focused on preventing HAIs in health workers. We then began the process of implementation and evaluation[26,28].

Meanwhile, PAHO invited our team to assist in preparing health workers for the Global Summit and the Pan American games in Trinidad and Tobago. The workplace audit tool, developed originally in Canada by the team (comprised of experts in program evaluation, infection control, occupational health, information technology, public health and medicine), and refined from use in Ecuador and South Africa, was again adapted and workshops held to train occupational health and infection control practitioners from 7 countries across the Caribbean. The audit tool is a structured form, which enables healthcare workers to evaluate their working environment in a systematic manner. Health and safety professionals have noted that the tool has enabled them to set priorities and act upon identified needs. A novel animated skill-building tool that permits health workers to practice selecting and wearing personal protective equipment was also developed for the Caribbean training (http://www.ghrpinnovation.com/ProtectPatti/Eng/index.html).

In collaboration with PAHO, the Basic Infection Control course originally developed in Canada post SARS was then translated into Spanish (http://www.ghrpinnovation.com/InfectionControl), with input from colleagues in Ecuador (led by co-author JB). We collaborated with member countries to pilot the online course in several countries to ensure its relevance to the local context. During the pilot phase, the participants expressed high levels of satisfaction towards the training specifically the interactive format and comprehensive content. The online course and tools, such as the workplace assessment, have been presented at various regional and national trainings such as the PAHO train-the-trainer workshops which were held in Venezuela, Colombia, Ecuador, Trinidad and Tobago and Belize. The Latin American and South African work also included development of evidence-based training programs to specifically build capacity of health and safety committees, as our previous research indicated was important [29]. The tools developed have since been revised and are now being used to train health and safety committees in Canada as well.

Advances in worker health and safety have been historically tied to workers’ struggles, led usually by trade unions, to obtain better working conditions. The well-being of the workforce, particularly when the economy is strained, as is occurring ever more forcefully in this era of deregulated globalization [4,30], is often treated as expendable by decision-makers. Ironically, perhaps, worker health and safety has not received greater attention in the healthcare sector than in other economic sectors[4], despite the fact that health workers constitute the largest workforce in the world, with an estimated 59 million worldwide [31]. While the tools we produced are limited in conveying an in-depth understanding of the complex global forces that weaken public health systems, hindering the allocation of resources to infection control and worker health, they do help mitigate the impact of resource strains in countries such as Ecuador and South Africa, where strong government commitment has been expressed towards health system improvement and worker well-being.

## Result outcomes

Our collaboration has produced a better understanding of the social, cultural, environmental, occupational and economic processes that determine the health of health workers locally [17,18,20,21,32-34] and globally[4,25,26,35]. Our conceptual framework has been since used by other research groups[36]; our findings were used by hospital decision-makers and government planners; and these research findings were taken into account by our own team in the development of the tools described above.

As noted above, the research we conducted first confirmed that providing health workers with training to properly protect themselves from infectious diseases is significantly associated with better perception of a positive safety climate. After we created training tools to address the organizational, environmental and individual factors we identified as important determinants of infection control compliance, we conducted further research following-up on the use of these online tools. We then found that providing time to take the course on work time was significantly associated with higher intention to comply with safety precautions compared to promoting the course on a voluntary basis (logistic regression model showed a statistically significant difference between supervisor-required and voluntary groups with respect to perceived importance of infection control in the workplace, the extent to which the facility ensures patient safety, and the extent to which the facility ensures staff safety)[19]. This led to the course becoming mandatory in British Columbia [22].

Building on the findings of our research in Canada, initial work in Ecuador, and our pilot study in South Africa (for example, poor staff knowledge on recapping of needles as well as the finding that more than half the respondents felt that they were not given guidance as to how to perform their jobs safely [26]), we collaborated with government officials in Ecuador, South Africa and the Caribbean to develop guidelines, policies and programmes. We also worked with international agencies to develop new policy guidelines [15]. Acting on our own research findings, we created further training materials, addressing not only basic infection control and how to don and doff personal protective equipment, but how to establish health and safety committees, inspect workplaces, investigate incidents, and establish policies and health and safety programs based on solid evidence.

Our work has squarely addressed North-South power relations and the digital divide, always building on local capabilities to transfer knowledge South-South, North-South and vice versa in a respectful manner that benefits both Northern as well as Southern partners [37]. The products developed have been widely heralded as innovative and important components of “toolkits” used internationally. The tools now used in Canada have, in turn, been improved from the collaborative adaptation process for South and Central America and South Africa.

Thus this research has resulted in health service approaches, products and policies that are being embraced nationally (e.g. in Ecuador, Trinidad-Tobago and South Africa) and internationally (e.g. through international organizations including PAHO) as well as having Canadian impact[19,21,22]. The guidelines, research and needs assessment instruments, web-based health information system, and on-line learning modules will continue to have widespread impact well into the future. More importantly, by elucidating the links between worker health and the health of patients, we have begun to show that attending to the health of the healthcare workforce is not only the right thing to do to protect this vulnerable population, but also produces safer healthcare for all. This case thus illustrates the benefits of infection control and occupational health researchers working together and also how Canadians and Southern partners alike benefit from international collaboration.

## The partnership

This case study is really about a partnership of partnerships. First, there was the partnership between Canadian occupational health and infection control [17-21,32,34] researchers, and simultaneously, a partnership between an inter-disciplinary Ecuadorean occupational and environmental health team and Canadian counterparts who shared an appreciation of an ecosystem approach to human health[38], including its applicability to emerging infectious diseases[39]. Meanwhile, a new partnership was being forged between the combined Canadian occupational health and infection control team and their South African counterparts [26], brought together by the World Health Organization (WHO). Finally, with the assistance of PAHO and later also the WHO, the various partnerships were brought together, informing each other in what became an integrated international approach to promoting healthy healthcare.

Knowledge translation experts emphasize the importance of good quality evidence[38] as well as involving users of the research findings at the earliest stage. Thus it was essential that we involved the local healthcare leadership, already established occupational health services as well as health and safety committee members and governmental-based expertise at the outset. In Ecuador, the project built on a strong partnership between the University of Andina Simon Bolivar, the University of Cuenca, and various other universities and healthcare facilities on one hand, and the various centres at the University of British Columbia on the other. Having a strong local champion is key to success, and Ecuadorean co-author (JB) fulfilled that role. Similarly, we chose Pelonomi Regional Hospital in the Free State as a research pilot site to support knowledge translation and capacity building, in large part due to the local champion.

A major impact of our work to date has been the demonstration of the benefits of close collaboration between infection control and occupational health, which, in most jurisdictions, was weak. The Director of the National Institute for Occupational Health in South Africa cited our collaboration as a model that should be embraced in South Africa. Linkages are now being fostered between infection control and occupational health personnel, modeled on the Canadian-initiated collaboration; OHASIS, or at least some modules from OHASIS, is being used by occupational health and infection control professionals and by health and safety committee members in Latin America, the Caribbean and South Africa, as are the interactive online training modules. The full benefits of these innovations will increasingly manifest over time, but the impact on knowledge, attitudes and practices has already begun to be demonstrated[25].

## Challenges and successes

Collaboration requires mutual respect and trust, as well as a shared vision and sense of common mission. We were fortunate that the various partnerships within this partnership-of-partnerships all agreed to an open source, creative commons philosophy, in which none of the products of our work would be commercialized. This viewpoint also maintains that all derivatives must be approved by all members of the collective, which ensures on-going quality improvement and a flexible, yet standardized, and more easily communicated approach. With this sense of common mission, we are confident that the fruits of our collaboration will continue to provide high quality knowledge transfer of best available evidence.

Our first real challenge was in sustaining engagement of politically active workplace stakeholders, specifically the trade unions. We have had decades of successful experience in this regard[29,39,40], but may have taken for granted that labour union trust would be there. While this was not problematic in our Latin American work, a communications breakdown occurred in the South African work, creating a setback. The lesson learned was that trust can never be assumed, and it is well worth taking the time to ensure that all key stakeholders are indeed engaged before the project moves ahead too far. Getting process issues right is paramount to success.

A second challenge, also stemming from politically-charged labour relations, was the advent of a major strike in South Africa just as we were beginning what was supposed to have been an intensive two-week capacity-building effort. The team therefore had to come up with training innovations (including role-play, drawn scenarios, and interactive on-line learning modules [19,21,22,41]); with necessity being the mother of invention, the products created were very well-received and will serve the cross-continental partnerships well for years to come.

Another important lesson to note is the importance of thinking about scale-up and sustainability from the start. While the Ecuadorean pilots were successful, resources are not in place to continue the efforts at the desired intensity. Learning from this, before launching the full pilot in South Africa, decision-makers (including co-author LN) started planning for scale-up early, should the pilot prove successful. This required thinking through complexities beyond the pilot, such as who will continue to implement and monitor the model after the pilot has ended, and how should the model be altered in the pilot with such questions in mind. By working closely with the WHO, the International Labour Office, the International Commission on Occupational Health, and a world-expert on scaling up[42], we are now optimistic that the tools produced will be successfully used not only in local pockets, but on national and international scale.

From a funding perspective, the increasingly embraced philosophy of open source[43] and creative commons licensing [44] assures that these tools are available without charge. Key however, will be the extent to which local (and national) colleagues are indeed comfortable in using the tools and promoting their use locally. Also, in the case of information technology that requires maintenance and periodic updating, commitment from authorities (either government or external funders/partners) is needed. The “business model” of using revenue from distribution in high-income countries to fund maintenance and updates globally, is one way that LMICs can have their systems maintained and updated without strain on their resources, and assist high income countries to assume their global responsibilities [35].

Finally, it should be stressed that the success of this work has been, and will continue to be, based on front-line support and active engagement of the decision-makers. This can never be taken for granted.

## Competing interests

No competing interests to declare.

## Authors' contributions

AY and EAB conceived these projects and created the first draft of this manuscript. JB was responsible for the coordination of the projects in Ecuador. NL was responsible for the projects in South Africa. MCL aided in the coordination of the projects in Venezuela and in Trinidad and Tobago. All authors (AY, EAB, JB, NL, MCL, KL and JS) helped to write and revise this manuscript.

## References

[B1] The World Health Report http://www.who.int/whr/2002/chapter4/en/index8.html

[B2] Chan-Yeung M Severe Acute Respiratory Syndrome (SARS) and health care workers Int J Occup Environ Health 2004 10 421 427 15702757 10.1179/oeh.2004.10.4.421

[B3] Staes C Wuthrich A Gesteland P Allison M Leecaster M Shakib J Carter M Mallin B Mottice S Rolfs R Public health communication with frontline clinicians during the first wave of the 2009 influenza pandemic J Public Health Manag Pract 2011 17 1 36 44 21135659 10.1097/PHH.0b013e3181ee9b29 PMC3070179

[B4] Spiegel JM Labonte R Ostry AS Understanding "globalization" as a determinant of health determinants: a critical perspective Int J Occup Environ Health 2004 10 Special Edition Spring 360 367 15702749 10.1179/oeh.2004.10.4.360

[B5] Schrecker T Labonte R Taming the 'brain drain': A challenge for public health systems in Southern Africa Int J Occup Environ Health 2004 Special Edition Spring 10.1179/oeh.2004.10.4.409 15702755

[B6] Bigelow P Moore D Yassi A Assessing the health implications for healthcare workers of regulatory changes eliminating locally developed occupational exposure limits in favor of TLVs: an evidence-based bipartite approach Int J Occup Environ Health 2004 10 433 444 15702759 10.1179/oeh.2004.10.4.433

[B7] International Air Transportation Association Fact sheet: IATA-International Air Transport Association 2009 Available from http://www.iataorg/pressroom/facts_figures/fact_sheets/iatahtm

[B8] Haley R Culver D White J Morgan W Emori T Munn V Hooton T The efficacy of infection surveillance and control programs in preventing nosocomial infection is US hospitals Am J Epidemiol 1985 121 2 182 205 4014115 10.1093/oxfordjournals.aje.a113990

[B9] Allegranzi B Pittet D Healthcare-associated infection in developing countries: simple solutions to meet complex challenges Infect Control Hosp Epidemiology 2007 28 1323 1337 10.1086/521656 17994510

[B10] Lazzari S Allegranzi B Concia E Making hospitals safer: the need for a global strategy for infection control in health care settings World Hosp Health Serv 2004 40 32 42 15338996

[B11] Harbarth S Saxa H Gastmeierb P The preventable proportion of nosocomial infections: an overview of published reports J Hosp Infect 2003 54 4 258 266 10.1016/S0195-6701(03)00150-6 12919755

[B12] James P Christopher DJ Balamugesh T Gupta R Death of a health care worker with nosocomial extensively drug-resistant tuberculosis in India Int J Tuberc Lung Dis 2009 13 6 795 796 19460260

[B13] Lacy MD Horn K Nosocomial transmission of invasive group a streptococcus from patient to health care worker Clin Infect Dis 2009 49 3 354 357 10.1086/599832 19580415

[B14] Pruss-Ustun A Rapiti E Hutin Y Sharps injuries: global burden of disease from sharps injuries to health-care workers WHO Environmental Burden of Disease Series 2003 3 10.1002/ajim.20230 16299710

[B15] Yassi A O’Hara L LoChang J Lockhart K Spiegel J Provision of Workplace Programs for HIV and TB for Healthcare Workers - A Systematic Review to support development of Guidelines for the Implementation of Policies to Improve Health Worker Access to Prevention, Diagnosis, Treatment, and Care Services for HIV and TB 2009 Vancouver: Prepared for the World Health Organization

[B16] O'Donnell MR Jarand J Loveday M Padayatchi N Zelnick J Werner L Naidoo K Master I Osburn G Kvasnovsky C High incidence of hospital admissions with multidrug-resistant and extensively drug-resistant tuberculosis among South African health care workers Ann Intern Med 2010 153 8 516 522 20956708 10.1059/0003-4819-153-8-201010190-00008 PMC3074259

[B17] Yassi A Lockhart K Copes R Kerr M Corbiere M Bryce E Danyluk Q Keen D Yu S Kidd C Determinants of healthcare workers' compliance with infection control procedures Healthc Q 2007 10 1 44 52 17326369 10.12927/hcq.2007.18648

[B18] Bryce E Copes R Gamage B Lockhart K Yassi A Staff perception and institutional reporting: two views of infection control compliance in British Columbia and Ontario three years after an outbreak of severe acute respiratory syndrome J Hosp Infect 2008 69 2 169 176 10.1016/j.jhin.2008.03.010 18485532 PMC7118975

[B19] Yassi A Bryce E Novak Lauscher H Maultsaid D Zhao K The impact of requiring completion of an on-line infection control course on health professionals’ intentions to comply with infection control guidelines: a comparative study Can J Infect Dis Med Microbiol 2009 20 1 15 19 20190890 10.1155/2009/879357 PMC2690520

[B20] Moore DM Gilbert M Saunders S Bryce E Yassi A Occupational health and infection control practices related to severe acute respiratory syndrome: health care worker perceptions AAOHN J 2005 53 6 257 266 16018538

[B21] Hon C Gamage B LoChang J Bryce E Yassi A Maultsaid D Yu S Personal protective equipment in healthcare: Can online infection control courses transfer knowledge and improve proper selection and use? Am J Infect Cont 2008 36 10 e33 37 10.1016/j.ajic.2008.07.007 PMC7132690 19084161

[B22] Bryce E Choi P Landstrom M LoChang J Using Online Delivery for Workplace Training in Healthcare J Dist Educ 2008 22 3 149 156

[B23] Fujii R Yassi A Tennassee M Arauz V Bryce E Lavoie M Healthy Hospitals Project Part I (Audit and Survey): Collaboration between Canada and Ecuador CARWH conference - Occupational Health and Safety Research in Action: Method, Results and Applications 2008 Montreal, Quebec

[B24] Fujii R Yassi A Tennassee M Cvitkovich Y Lavoie M Healthy Hospitals Project Part II (Workshop): Collaboration between Canada and Ecuador CARWH conference - Occupational Health and Safety Research in Action: Method, Results and Applications 2008 Montreal, Quebec

[B25] Lavoie M-C Yassi A Bryce E Fujii R Longronio M Tennassee M International collaboration to protect health workers from infectious diseases PAHO Journal 2010 27 5 396 402 10.1590/s1020-49892010000500010 20602074

[B26] Yassi A Bryce E Spiegel J Building capacity to secure healthier and safer working conditions for healthcare workers: A South African-Canadian collaboration Int J Occup Environ Health 2009 15 4 360 369 19886346 10.1179/oeh.2009.15.4.360

[B27] Spiegel JM Lockhart K LoChang J Tremblay J Dybka L Yassi A Workplace and workforce health information systems in healthcare: Acknowledging the role of university researchers and highlighting the importance of health and safety committee capacity-building Can J Public Health 2009 100 2 157 19839296 10.1007/BF03405528 PMC6973659

[B28] Spiegel J Yassi A Rees D Kielkowski D Ross M Ndelu L Tool, Weapon or White Elephant? A Canadian-South African Collaboration to explore how information systems can be harnessed to help make health care a healthier place to work American College of Occupational and Environmental Medicine, International Commission on Occupational Health 2007 Vancouver, BC, Canada

[B29] Yassi A Ostry AS Hatter B De Boer HM Joint health and safety committee education and the value of bipartite cooperation in the healthcare sector in British Columbia, Canada Int J Occup Environ Health 2005 11 3 305 312 16130973 10.1179/107735205800246019

[B30] Labonte R Schrecker T Globalization and social determinants of health: The role of the global marketplace (part 2 of 3) Globalization and Health 2007 3 6 10.1186/1744-8603-3-6 PMC1919362 17578569

[B31] World Health Organization World Health Report 2006: Working Together For Health 2006 http://www.who.int/hiv/pub/meetingreports/TTRmeetingreport2.pdf [homepage on the internet]

[B32] Gamage B Moore D Copes R Yassi A Bryce E Group TBIRP Protecting health care workers from SARS and other respiratory pathogens: a review of the infection control literature Am J Infect Control 2005 33 2 114 121 10.1016/j.ajic.2004.12.002 15761412 PMC7132691

[B33] Moore D Gamage B Bryce E Copes R Yassi A Protecting health care workers from SARS and other respiratory pathogens: organizational and individual factors that affect adherence to infection control guidelines Am J Infect Control 2005 33 2 88 96 10.1016/j.ajic.2004.11.003 15761408 PMC7115321

[B34] Yassi A Moore D Fitzgerald JM Bigelow P Hon CY Bryce E Research gaps in protecting healthcare workers from SARS and other respiratory pathogens: an interdisciplinary, multi-stakeholder, evidence-based approach J Occup Environ Med 2005 47 1 41 50 15643158 10.1097/01.jom.0000150207.18085.41 PMC4880470

[B35] Yassi A Bryce E Spiegel J Assuming our global responsibility: Improving working conditions for healthcare workers globally Open Medicine 2009 3 3 174 177 PMC3090118 21603046

[B36] Nichol K Bigelow P O'Brien-Pallas L McGeer A Manno M Holness D The individual, environmental, and organizational factors that influence nurses' use of facial protection to prevent occupational transmission of communicable respiratory illness in acute care hospitals Am J Infect Control 2008 36 7 481 487 10.1016/j.ajic.2007.12.004 18786451 PMC7132646

[B37] Bryce E Yassi A Nophale L Scharf S Dybka L Lavoie M-C Tennassee M Fujii R International adaptation and sharing of an infection control and occupational health inspection tool for healthcare facilities: Bringing lessons back home International Commission on Occupational Health Congress 2009 Cape Town, South Africa

[B38] Grimshaw J Santesso N Cumpston M Mayhew A McGowan J Knowledge for knowledge translation: The role of the Cochrane Collaboration J Contin Educ Health Prof 2006 26 1 55 62 10.1002/chp.51 16557512

[B39] Yassi A The development of worker-controlled occupational health centers in Canada Am J Public Health 1988 78 6 689 693 10.2105/AJPH.78.6.689 3369601 PMC1350285

[B40] Yassi A Ostry AS Spiegel J Walsh G de Boer HM A collaborative evidence-based approach to making healthcare a healthier place to work Hosp Q 2002 5 3 70 78 12055871 10.12927/hcq.2002.16759

[B41] O’Hara L Bryce E Scharf S Yassi A Innovative training for occupational health and infection control workplace assessment in healthcare: Necessity as the mother of invention Am J Health Educ 2011 accepted for publication

[B42] Kohl R Implementing best practices in reproductive health: A guide for fostering change to scale up effective health services 2007

[B43] UBC UILO Global access http://www.uilo.ubc.ca/about/initiatives/global.html Retrieved March 7 2011

[B44] Creative Commons licensing http://creativecommons.org Retrieved March 8 2011

